# CD38 Inhibition Ameliorates Age-Related CognitiveDecline via a Choroid Plexus–Cerebrospinal Fluid–Hippocampus Axis

**DOI:** 10.21203/rs.3.rs-8330519/v1

**Published:** 2026-01-06

**Authors:** Eric Verdin, Jingqi Fang, Rebeccah Riley, Kevin Schneider, Rosalba Perrone, Prasanna Kumaar, Christina King, Grant Kauwe, Andrea Roberts, Genesis Vega Hormazabal, Yini Zhang, Ethan Millard, Xinran Liu, Wendy Jara, Carlos Galicia Aguirre, Harrison Baker, Natalia Murad, Ben Ambrose, Tommy Tran, Nicolas Martin, Ran Zhang, Durai Sellegounder, Simon Melov, David Furman, Tara Tracy, Akos Gerencser, Birgit Schilling, Lisa Ellerby

**Affiliations:** Buck Institute for Research on Aging; Buck Institute for Research on Aging; Buck Institute Research on Aging; Buck Institute; Buck Institute for Research on Aging; Buck Institute for Research on Aging; Buck Institute for Research on Aging; Buck Institute for Research on Aging; Buck Institute for Research on Aging; Buck Institute for Research on Aging; Buck Institute for Research on Aging; Buck Institute for Research on Aging; Buck Institute for Research on Aging; Buck Institute for Research on Aging; Buck Institute for Research on Aging; Buck Institute for Research on Aging; Buck Institute for Research on Aging; Buck Institute for Research on Aging; Buck Institute for Research on Aging; Buck Institute for Research on Aging; Buck Institute for Research on Aging; Buck Institute for Research on Aging; Buck Institute for Research on Aging; Buck Institute; Buck Institute; Buck Institute for Research on Aging; Buck Institute for Research on Aging

## Abstract

Age-related cognitive decline represents a major and unresolved challenge of human aging. Here, we identify the NAD^+^-consuming enzyme CD38 as a central regulator of cognitive aging acting through a choroid plexus–cerebrospinal fluid (CSF)–hippocampus axis. CD38 expression increases with age and localizes primarily to pericytes in the choroid plexus, where it depletes NAD^+^, impairs mitochondrial function, and promotes cellular senescence. Genetic ablation or pharmacological inhibition of CD38 restores NAD^+^ levels, suppresses senescence markers, and improves choroid plexus function, resulting in a rejuvenated CSF proteomic and metabolomic profile characterized by reduced inflammatory signaling and enhanced neurotrophic support. These changes propagate to the hippocampus, reversing age-related transcriptional signatures and enhancing synaptic plasticity. A novel, brain-penetrant CD38 inhibitor, NTX-748, reproduced the benefits of CD38 deficiency—elevating systemic and brain NAD^+^ levels, improving long-term potentiation, and enhancing multiple domains of cognition in aged mice. Collectively, these findings identify the choroid plexus as a metabolic gatekeeper of brain aging and establish CD38 inhibition as a promising therapeutic strategy to promote cognitive resilience and healthy brain aging.

Cognitive decline is one of the most devastating manifestations of aging, affecting millions of individuals worldwide and imposing substantial socioeconomic burdens^1,2^. The mechanisms underlying age-related cognitive impairment are complex and multifaceted and involve the progressive deterioration of cellular function across multiple brain regions and support systems^3,4^. Central to this process is the age-associated depletion of nicotinamide adenine dinucleotide (NAD^+^), an essential coenzyme that participates in over 500 enzymatic reactions^5^. NAD^+^ serves critical roles in energy metabolism, DNA repair, epigenetic regulation, and the activity of NAD^+^-dependent enzymes, including sirtuins and poly(ADP-ribose) polymerases^6–9^. The decline in NAD^+^ levels during aging has been causally linked to multiple hallmarks of aging, such as mitochondrial dysfunction, cellular senescence, compromised proteostasis, and altered intercellular communication^6,10–13^.

The discovery that CD38, a transmembrane NAD^+^ glycohydrolase, drives age-related NAD^+^ depletion has provided crucial mechanistic insights into metabolic aging^14^. CD38 is a multifunctional enzyme that depletes cellular NAD^+^ pools through its NAD^+^ glycohydrolase activity, which hydrolyzes NAD^+^ to nicotinamide (NAM) and adenosine diphosphate-ribose (ADPR), and its ADP-ribosyl cyclase activity, which converts NAD^+^ to cyclic ADPR (cADPR), with both ADPR and cADPR serving as calcium-mobilizing second messengers^15,16^. CD38 expression and activity increase dramatically with age across multiple tissues, and CD38 knockout mice maintain youthful NAD^+^ levels and are protected from age-related mitochondrial dysfunction via a SIRT3-dependent mechanism^14^. Supporting the therapeutic potential of targeting CD38, pharmacological inhibition with the small molecule 78c extends median lifespan by 17% in male mice and improves exercise performance, endurance, and metabolic function in naturally aged animals^17^. Senescent cells promote tissue CD38^+^ expression in macrophages through the senescenceassociated secretory phenotype (SASP). These findings establish that SASP factors induce macrophage proliferation and CD38 expression, creating an expanding population of NAD^+^-consuming cells that accelerates metabolic dysfunction during aging^18,19^. However, the specific cellular sources of CD38 in the brain and the mechanisms by which CD38 influences normal cognitive decline are poorly understood.

The choroid plexus has emerged as a critical but historically understudied regulator of brain homeostasis and cognitive function^20–22^. This highly vascularized epithelial structure extends into each brain ventricle, where it produces cerebrospinal fluid (CSF) at a rate of 400–600 mL per day in humans^23^. Beyond its classical role in CSF production, the choroid plexus functions as a selective blood-CSF-barrier and secretes a complex array of signaling molecules that influence brain development and function^24–26^. The CSF provides a proliferative niche for neural progenitor cells^27^, with age-dependent effects on neurogenesis that are largely attributable to growth factors^27^. The CSF proteome is elaborate, dynamic, and contains hundreds of factors that coordinate brain development and maintenance^28^. Spatial and temporal heterogeneity adds another layer of complexity to choroid plexus function. Single-cell analyses have further delineated the cellular diversity within the choroid plexus, identifying distinct populations of epithelial cells, pericytes, fibroblasts, and immune cells^29^.

During aging, the choroid plexus undergoes epithelial atrophy, basement membrane thickening, and reduced CSF production by up to 50%^30–32^. These structural changes are accompanied by transcriptional alterations, with aged choroid plexus showing upregulation of inflammatory and cellular stress markers^33^. Recent neuroimaging studies have shown enlarged choroid plexus volumes in patients with AD or with mild cognitive impairment, the volume changes correlate with cognitive performance and disease severity^34,35^. The choroid plexus also serves as a neuroimmune interface, with age-related accumulation of immune cells contributing to neuroinflammation^36^. Despite growing evidence linking choroid plexus dysfunction to cognitive decline, the molecular mechanisms driving these age-related changes remain poorly characterized.

The intersection of NAD^+^ metabolism, CD38 activity, and choroid plexus function is not understood during brain aging. The choroid plexus exhibits exceptionally high metabolic activity and serves as a critical interface between the systemic circulation and the brain^26,37^. Thus, it may be particularly vulnerable to age-related metabolic perturbations. Moreover, the strategic position of the choroid plexus as the primary source of CSF means that metabolic dysfunction in this tissue could have widespread effects throughout the brain via alterations in CSF composition. Here, we identify CD38 in choroid plexus pericytes as a critical regulator of cognitive aging through modulation of a choroid plexus–CSF–hippocampus axis.

## RESULTS

### CD38 expression increases in brain aging, and its deficiency enhances cognitive performance

**Figure 1:**
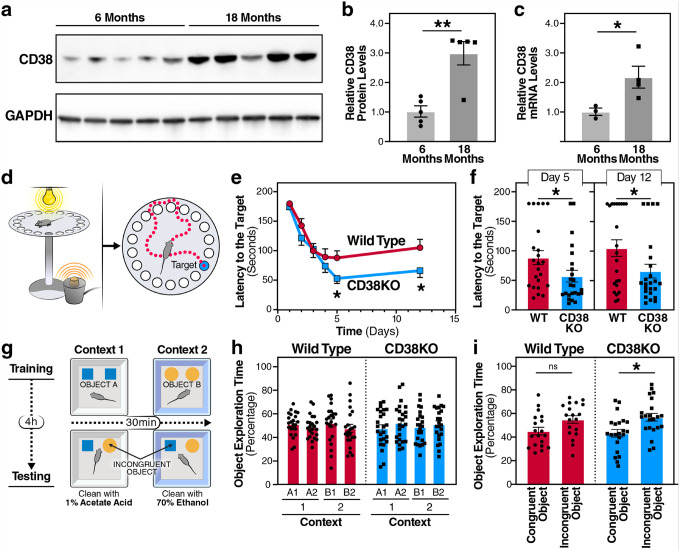
CD38 expression increases with age, and CD38 knockout enhances cognitive function across multiple behavioral paradigms. **a,** Representative western blot analysis showing CD38 protein expression in whole-brain lysates from mice at 6 and 18 months of age (n=5 per group). GAPDH served as a loading control. **b,** Quantification of CD38 protein levels normalized to 4-month values (n=5 per group). Data represent mean ± s.e.m., **P<0.01 by unpaired t-test. **c,** Quantification of *Cd38* mRNA levels normalized to 6-month values. Data represent mean ± s.e.m. *P<0.05 by unpaired t-test. **d,** Schematic of the Barnes maze spatial learning and memory test. Mice learn to locate a target escape hole among 40 holes arranged in a circle on an elevated platform using distal spatial cues. **e,** Learning curves showing latency to locate the target hole during training in 18-month-old wild-type (WT) and CD38 knockout (CD38KO) mice. CD38KO mice learn faster than age-matched WT controls. Data represent mean ± s.e.m. *P<0.05 by unpaired t-test. **f,** Comparison of target-finding latency during short- and long-term memory testing phases. CD38KO mice showed significantly reduced latency at both time points on day 5 and day 12, indicating enhanced spatial memory consolidation and retention. Data represent mean ± s.e.m. Individual data points are shown. *P<0.05 by unpaired t-test. **g,** Schematic representation of the pattern separation behavioral paradigm. Mice were trained to distinguish between two similar contexts (Context 1 and Context 2) differing in visual, tactile, and olfactory cues. After 4 hours of training, mice were tested for their ability to discriminate between the congruent and incongruent objects in each context. **h,**Exploration time was quantified during the training phase of the object context discrimination task. The 18-month-old WT and CD38KO mice spent a similar amount of time exploring the objects in the two contexts during the training phase. Object exploration time is expressed as a percentage. Data represent mean ± s.e.m. **i,** Summary analysis of pattern separation memory performance during the test phase of the object context discrimination task comparing congruent vs incongruent object exploration between WT and CD38KO mice. CD38KO mice explored the incongruent object significantly more than the congruent object. Data represent mean ± s.e.m. Individual data points are shown. *P<0.05 by paired t-test; ns, not significant.

The decline in NAD^+^ levels is a hallmark of cellular aging, yet the specific mechanisms driving NAD^+^ depletion in the brain are not completely understood^38^. We hypothesized that CD38, the primary NAD^+^-consuming enzyme in mammalian tissues^39^, contributes to age-related cognitive decline. To evaluate this hypothesis, we first characterized CD38 expression patterns with 6-month-old and 18-month-old murine brain tissue. Western blot analysis of whole-brain lysates revealed a striking increase in CD38 protein expression (Fig. 1a,b). CD38 levels increased when comparing young adult (6-month-old) to aged (18-month-old) mice. Consistent with the protein levels, *Cd38* mRNA also increased in 18-month-old mice compared to the 6-month-old mice (Fig. 1c). This progressive upregulation parallels previous observations of CD38 accumulation in other tissues during aging^14^ and suggests that CD38 may contribute to age-related brain dysfunction.

To directly assess the functional consequences of CD38 expression on cognitive performance in aged mice, we subjected 18-month-old wild-type (WT) and CD38 knockout (CD38KO) mice to a comprehensive battery of cognitive tests. In the Barnes maze, a measurement of hippocampal-dependent spatial learning and memory, CD38KO mice performed better than age-matched WT controls (Fig. 1d–f). During the acquisition phase, CD38KO mice showed accelerated learning curves with progressively shorter latencies to locate the escape hole when compared to WT mice (Fig. 1e). The enhanced learning translated into robust memory retention, with CD38KO mice showing significantly better performance in short- and long-term probe trials than WT controls (Fig. 1f).

The cognitive benefits of CD38 deficiency extended beyond spatial navigation. To evaluate cognitive flexibility, we used a pattern separation task (Fig. 1g–i). In this paradigm, mice were trained to differentiate between two contexts with distinct visual, tactile, and olfactory cues and then tested for their ability to discriminate between congruent and incongruent object-context pairings. During the sample phase of the object-context discrimination test of pattern separation memory, the mice spent equivalent time exploring the distinct pairs of identical objects in two different but similar contexts. During the memory test phase, one of the objects in each pair is switched between contexts (Fig. 1g). While 18-month-old WT mice showed limited discrimination between congruent and incongruent objects, the age-matched CD38KO mice exhibited a statistically significant increase in exploration of the incongruent object, demonstrating enhanced pattern separation memory (Fig. 1h,i). These findings establish that genetic ablation of CD38 confers cognitive benefits in aged mice, encompassing both spatial and pattern separation memory, suggesting that age-related CD38 upregulation is a targetable driver of age-associated cognitive decline.

### CD38 localizes to the choroid plexus pericytes

**Figure 2:**
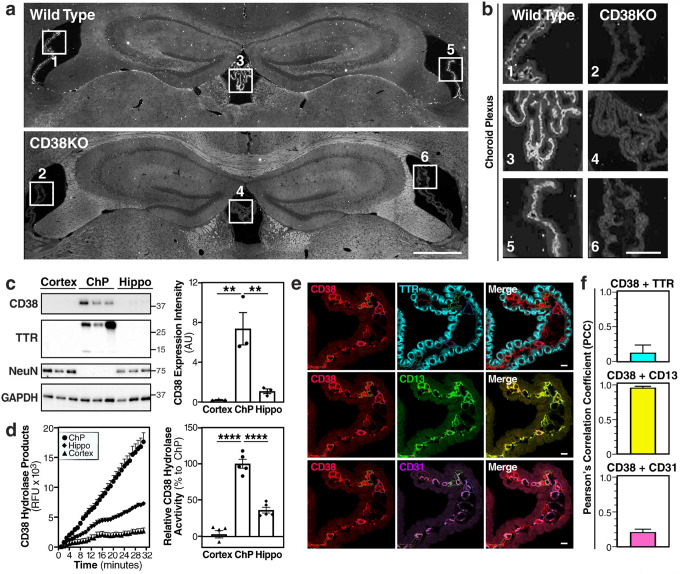
CD38 is predominantly expressed in the choroid plexus and localizes to pericytes. **a,** Coronal brain sections showing CD38 immunofluorescence staining in wild-type (WT, top) and CD38 knockout (CD38KO, bottom) mice. Red dashed boxes indicate choroid plexus regions with robust CD38 expression in lateral ventricles (boxes 1, 5) and third ventricle (box 3) in WT mice, with complete absence of signal in CD38KO mice (boxes 2, 4, 6). Scale bar, 500 μm. **b,** Higher magnification images of choroid plexus regions corresponding to numbered boxes in panel a, comparing CD38 immunoreactivity between WT and CD38KO mice. A strong CD38 signal is observed in WT choroid plexus structures across different ventricular locations, whereas CD38KO mice show no detectable signal, confirming antibody specificity. Scale bar, 100 μm. **c,** Left: Western blot analysis of CD38 expression across different brain regions (cortex, choroid plexus (ChP), hippocampus) showing the highest expression in choroid plexus, compared to cortex and hippocampus. TTR (transthyretin) serves as a choroid plexus marker, NeuN confirms neuronal tissue presence, and GAPDH serves as a loading control. Right: Quantification of CD38 protein expression intensity across brain regions, demonstrating significantly higher CD38 levels in the choroid plexus than cortex and hippocampus. Data represent mean ± s.e.m. **P<0.01 by one-way ANOVA with post-hoc analysis. **d,** Left: CD38 hydrolase enzymatic activity assay showing differential activity over time in different brain tissues, including ChP, hippocampus (Hippo), and cortex. Right: Relative NAD^+^ hydrolase activity quantification. Data represent mean ± s.e.m. (n=5–6). ****P<0.0001 by one-way ANOVA. **e,** Co-immunofluorescence staining of choroid plexus showing CD38 (red) expression with different cellular markers. Top: CD38 and TTR (epithelial marker, cyan); middle: CD38 and CD13 (pericyte marker, green); bottom: CD38 and CD31 (endothelial marker, purple). Merged images demonstrate CD38 predominantly localizes to pericytes (CD13-positive) rather than epithelial or endothelial cells. Scale bar, 10 μm. **f,** Quantification of colocalization between CD38 and different cellular markers by Pearson’s correlation coefficients, confirming the highest colocalization with CD13 (pericyte marker). n=5, Data represent mean ± s.e.m.

Next, we evaluated the expression and localization of CD38 in the aging brain. Immunofluorescence mapping revealed an unexpected distribution pattern: CD38 expression was notably sparse in hippocampal and cortical regions, the areas traditionally associated with learning and memory. Instead, we observed intense CD38 immunoreactivity specifically within the choroid plexus structures of all brain ventricles—lateral, third, and fourth—with complete absence of signal in CD38KO mice, confirming antibody specificity (Fig. 2a,b).

Biochemical validation across different brain regions confirmed this localization pattern. Western blot analysis demonstrated that CD38 protein levels in isolated choroid plexus are 36.5 and 6.9-fold higher than in the cortex and hippocampus, respectively. Transthyretin (TTR) confirmed the enrichment of our lysates for choroid plexus marker with no TTR expression in the hippocampus or cortex (Fig. 2c). This regional specificity was further corroborated by enzymatic activity assays, where choroid plexus tissue had 35.7 and 2.7-fold greater NAD^+^ hydrolysis activity than cortical and hippocampal tissue, respectively (Fig. 2d).

The choroid plexus consists of epithelial cells responsible for CSF production, endothelial cells forming the vasculature, and pericytes that regulate vascular function^20,21^. To identify the specific cell-type that expressed CD38 within the choroid plexus, we performed high-resolution co-immunofluorescence staining with markers for pericytes, epithelial, and endothelial (Fig. 2e). CD38 showed minimal colocalization with TTR (a marker of choroid plexus epithelial cells) or CD31 (a marker of endothelial cells). Remarkably, CD38 exhibited near-complete colocalization with CD13 (aminopeptidase N, a specific marker of pericytes) (Fig. 2f). Three-dimensional reconstruction of confocal z-stacks confirmed that CD38 expression was restricted to the pericyte layer wrapping around the choroid plexus vasculature (**Supplementary Fig. 1**). The predominant expression of CD38 in choroid plexus pericytes, rather than in neurons, suggests that CD38 may influence cognitive function by modulating the brain’s cerebrospinal fluid.

### CD38 deficiency alters NAD^+^ metabolism, enhances mitochondrial respiration, and reverses cellular senescence signatures in choroid plexus

**Figure 3:**
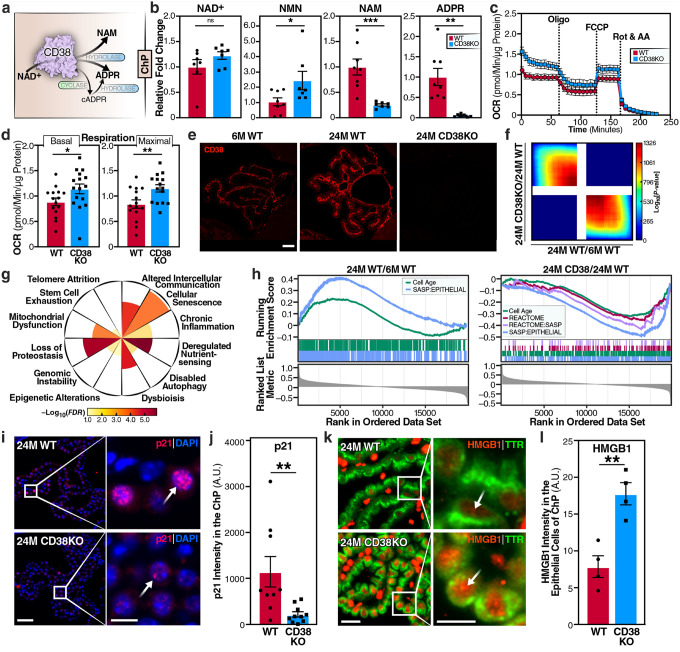
CD38 loss preserves NAD^+^–related metabolites, enhances mitochondrial respiration, and attenuates senescence programs in the choroid plexus. **a,** Schematic of CD38-catalysed conversion of NAD^+^ to nicotinamide (NAM), ADP-ribose (ADPR), and cyclic ADPR (cADPR). **b,** NAD^+^ and related metabolites in choroid plexus from 18-month-old mice (n=6–8 per group). **c,** Oxygen consumption rate (OCR) traces from choroid plexus of 18-month-old mice with sequential additions of oligomycin, FCCP, and rotenone/antimycin A. **d,** Basal and maximal respiration quantified from **c**. **e,** CD38 immunofluorescence (red) in choroid plexus from young (6-month) and aged (24-month) WT mice, and aged CD38KO mice. **f,** Discordance between aging (23 M WT/6M WT) and CD38KO (24M CD38KO/24M WT) using a rank–rank hypergeometric overlap (RRHO); color scale indicates −log_10_(adjusted *P* value). **g,** Enrichment analysis polar plot of aging hallmarks in genes significant (FDR <0.05) and discordant between aging and CD38KO; radial length, number of genes; color, −log_10_(false discovery rate, FDR). **h,** GSEA enrichment plots for CellAge and SASP:Epithelial gene sets comparing aged WT (24-month) versus young WT (6-month) (left) and aged CD38KO (24-month) versus aged WT (24 months) (right). **i,** p21 (red) and DAPI (blue) staining in 24-month choroid plexus; arrows indicate p21^+^ cells. Scale bars, 50 μm (left) and 10 μm (right). **j,** Quantification of p21 intensity from **i**. Data are mean ± s.e.m.; **P<0.01 by two-tailed unpaired t-test. (n=9–10 sections were obtained from 5 mice per group). **k,** HMGB1 (red) co-stained with transthyretin (TTR; green) in 24-month choroid plexus; arrows indicate HMGB1^+^ cells. Scale bars, 20 μm (left) and 10 μm (right). **l,** Quantification of HMGB1 intensity in epithelial cells from **k**. Data are mean ± s.e.m.; **P<0.01 by two-tailed unpaired t-test (n=4 sections were obtained from 4 mice per group).

To address how CD38 affects metabolism, we carried out targeted metabolomics. CD38 functions as the primary NAD^+^-consuming enzyme, converting NAD^+^ to nicotinamide (NAM), adenosine diphosphate-ribose (ADPR), and cyclic ADPR (cADPR)^40^ (Fig. 3a). Mass spectrometry analysis of choroid plexus tissue revealed a striking NAD^+^-related metabolic reprogramming in CD38KO mice (Fig. 3b). While NAD^+^ levels modestly increased, its precursor nicotinamide mononucleotide (NMN), which can also serve as alternative CD38 substrate^18,41^, was significantly increased. Conversely, the CD38 enzymatic products—NAM and ADPR—were markedly reduced, confirming the loss of CD38 enzymatic activity and consequent preservation of the NAD^+^ metabolites pool (Fig. 3b). Because cADPR is a low-abundance secondary metabolite, its levels in the choroid plexus were below the detection threshold in our analyses.

Although the elevation of NAD^+^ levels in choroid plexus was relatively modest, NAD^+^ is a central metabolic cofactor whose availability is tightly coupled to the activity of NAD^+^-dependent enzymes such as sirtuins, PARPs, and dehydrogenases. Additionally, the relationship between NAD^+^ availability and mitochondrial function is governed by enzyme kinetics rather than simple thermodynamics, meaning that even modest changes in NAD^+^ levels can significantly affect reaction rates and flux through oxidative phosphorylation pathways. Real-time measurement of oxygen consumption rate (OCR) with freshly collected choroid plexus revealed that CD38 deficiency significantly enhanced mitochondrial function (Fig. 3c,d). Both basal respiration and maximal respiratory capacity following FCCP-induced uncoupling were substantially increased in CD38KO choroid plexus compared to WT controls.

Immunofluorescence imaging confirmed robust CD38 expression in choroid plexus from 6-month and 24-month-old WT mice, with increased expression with age, and complete absence in CD38KO mice, confirming genotype specificity (Fig. 3e). Transcriptomic analysis using GeoMx spatial RNA sequencing revealed the gene expression signatures of CD38KO (24-month-old CD38KO vs 24-month-old WT) and aging (24-month-old WT vs 6-month-old WT) mice (**Supplementary Tables 1, 2**). Comparative analysis revealed overall discordance between the CD38KO and aging signatures (Fig. 3f), with 107 genes upregulated and 2 downregulated (FDR < 0.05; Supplementary Table 2), indicating that CD38 deletion broadly counteracts aging-associated transcriptional programs in the choroid plexus. Among these 109 enriched genes, we examined gene sets representative of the twelve canonical hallmarks of aging^4,4,42^ to identify the most affected pathways. Among them, the ‘cellular senescence’ hallmark showed the highest enrichment (Fig. 3g). Additionally, the senescence gene sets CellAge and SASP signatures were positively enriched in the aging signature and negatively enriched in the CD38KO signature, indicating that CD38 loss attenuates these age-associated senescence/SASP pathways (Fig. 3h).

Given the strong senescence transcriptomic signature, we examined p21 protein expression by immunofluorescence. We observed a reduction in p21-positive cells in aged CD38KO choroid plexus, compared to aged WT controls (Fig. 3i). In aged WT mice, p21-positive cells formed distinct clusters, suggestive of a paracrine senescence mechanism, whereas CD38KO tissue displayed only rare, isolated p21-positive cells (Fig. 3j). To identify which cell type was primarily affected by senescence, we performed co-immunofluorescence with specific cell-type markers. p21-positive cells predominantly colocalized with E-cadherin (**Supplementary Fig. 2**), identifying them as epithelial cells, the primary functional units responsible for CSF production. To confirm the hypothesis that epithelial cells were the primary site of senescence, we investigated the senescence marker HMGB1. HMGB1 secretion from senescent cells is a key driver of inflammatory signaling^43^. We used TTR as an epithelial marker to gate TTR^+^ regions of interest and measured HMGB1 signal exclusively within the epithelial region of interest (ROIs). Co-immunofluorescence revealed marked differences in epithelial HMGB1 localization under different conditions. In aged CD38KO epithelial cells, nuclear HMGB1 was preserved in contrast to WT (Fig. 3k,l). Interestingly, although CD38 expression is largely confined to choroid plexus pericytes, the observed epithelial-specific senescence implies a functional crosstalk between pericytes and epithelial cells. Loss of CD38 may thus preserve epithelial integrity by modulating pericyte-derived metabolic or signaling cues that sustain NAD^+^ availability and barrier function.

### Loss of CD38 reshapes CSF composition and preserves blood–CSF barrier integrity

**Figure 4:**
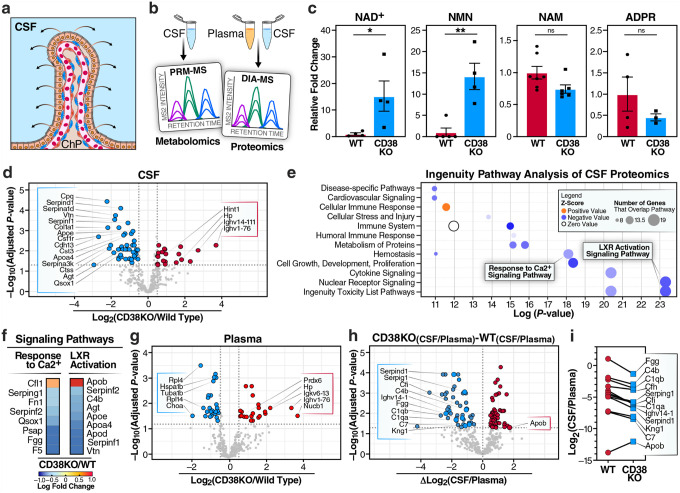
CD38 loss remodels the CSF proteome and NAD^+^ metabolism. **a,** Schematic illustration of the choroid plexus-CSF interface. **b,** Experimental workflow for comprehensive molecular profiling: targeted NAD^+^ metabolomics and untargeted proteomics analysis of CSF and plasma samples. PRM: Parallel Reaction Monitoring; DIA: Data-Independent Acquisition. **c,** Quantification of NAD^+^ and related metabolites (NMN, NAM, ADPR) in CSF from wild-type (WT) and CD38 knockout (CD38KO) mice measured by LC-MS/MS (n = 3–7 per group). Data represent mean ± s.e.m.; *P<0.05, **P<0.01 by two-tailed unpaired t-test; ns, not significant. **d,** Volcano plot displaying differential protein expression in CSF between 18-month-old CD38KO and WT mice (n=9 per genotype). Horizontal dashed line indicates adjusted *P*=0.05; proteins significantly upregulated (red) or downregulated (blue) in CD38KO are highlighted. **e,** Ingenuity Pathway Analysis (IPA) of CSF proteomics, revealing pathway category changes. Two prominently deactivated pathways in CD38KO mice are highlighted: “Response to Ca2^+^ Signaling Pathway” and “LXR Activation Signaling Pathway”. **f,** Heatmaps showing protein expression patterns of individual proteins comprising the Ca2^+^ signaling and LXR activation pathways identified in panel **e**. **g,** Volcano plot of differential protein expression in plasma between 18-month-old CD38KO and WT mice (n=9 per genotype). Horizontal dashed line indicates adjusted *P*=0.05; significantly altered proteins marked as in panel **d**. **h,** Volcano plot analyzing changes in blood-CSF barrier selectivity, calculated as CD38KO-WT (Δlog2(CSF/Plasma)) for all detected proteins. Horizontal dashed line indicates adjusted *P*=0.05; proteins with significantly altered barrier permeability shown in red (increased) or blue (decreased). **i,**12 proteins showing significant barrier selectivity changes, categorized into 6 functional groups based on their plasma enrichment patterns. Lines connect WT and CD38KO values for each protein.

The choroid plexus serves as the primary source of CSF (Fig. 4a), providing essential nutrients, growth factors, and signaling molecules to the brain^23^. To determine whether CD38 deficiency affects CSF composition, we performed targeted NAD^+^-related metabolomics and proteomics (data-independent acquisition mass spectrometry [DIA-MS]) of paired CSF and plasma samples from 18-month-old mice (Fig. 4b). CSF metabolite analysis revealed significantly elevated NAD^+^ and NMN levels in CD38KO mice, while NAM and ADPR levels trended lower but not significantly (Fig. 4c). Proteomic profiling of CSF identified numerous differentially abundant proteins (FDR <.05) between CD38KO and WT mice, Notably, several members of the serpin family (Serpind1, Serpina1d, and Serpina3k), which are established markers of neuroinflammation and astrocyte reactivity in aging and neurodegenerative diseases, were significantly downregulated in CD38KO CSF. This reduction in inflammatory serpins suggests that CD38 deletion attenuates age-related neuroinflammation in the CSF (Fig. 4d, **Supplementary Tables 3–8**). Ingenuity Pathway Analysis (IPA) shows significant alterations in pathway categories directly linked to CD38 function (Fig. 4e). Deactivation of the “Response to Ca^2+^ Signaling Pathway” in CD38KO CSF is consistent with the loss of CD38-mediated production of calcium-mobilizing second messengers ADPR and cADPR, which normally regulate intracellular calcium release^44^ and LXR activation (Fig. 4e). We observed deactivation of the “LXR Activation Signaling Pathway”, which is logical given that LXR (Liver X Receptor) acts upstream of CD38 and controls transporter-mediated cholesterol efflux—processes predicted to decline when LXR signaling is reduced^45^ (Fig. 4e). Specific genes that are altered in these two pathways are denoted in Fig. 4f. To assess whether these CSF changes reflected altered blood-CSF barrier function or selective secretion, we performed proteomic analysis of paired plasma samples from the same animals. The plasma proteome had fewer differentially abundant proteins compared to CSF (FDR <0.05; Fig. 4g) when comparing CD38KO and WT samples. Notably, analysis of CSF/plasma ratios revealed increased barrier selectivity in CD38KO mice (Fig. 4h,i) in comparison to aged WT mice. Most reduced proteins in the CSF versus plasma of CD38KO vs WT, included key plasma proteins such as fibrinogen gamma chain (Fgg), complement components (C4b, C1qb), coagulation factors (Cfh), and serpins (Serping1, Serpind1). This predominantly unidirectional change—with most proteins showing reduced CSF accumulation relative to plasma—suggests that removal/inhibition of CD38 may enhance barrier restrictiveness or alter active transport mechanisms at the blood-CSF interface, contributing to the distinct CSF proteome observed in CD38KO mice. These findings demonstrate that CD38 deficiency remodels both the CSF NAD^+^ metabolism and proteome.

### CD38 deficiency reverses aging signatures in the hippocampus

**Figure 5:**
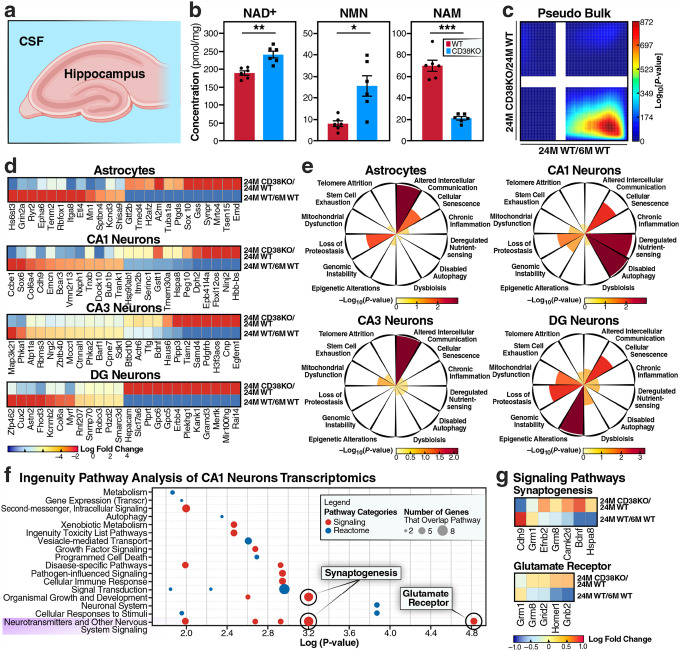
CD38 deficiency enhances hippocampal NAD^+^ metabolism and reverses age-related transcriptional signatures across multiple cell types. **a**, Schematic illustration showing the anatomical structure of the brain. **b,** Targeted metabolomic analysis of NAD^+^-related metabolites in hippocampal tissue. Quantification of NAD^+^, nicotinamide mononucleotide (NMN), and nicotinamide (NAM) concentrations in wild-type (WT) and CD38 knockout (CD38KO) mice (n=6–8 per group). Data presented as mean ± s.e.m.; *P<0.05, **P<0.01, ***P<0.001 by two-tailed unpaired t-test. **c,** Rank-rank hypergeometric overlap (RRHO) analysis of pseudo-bulk hippocampal transcriptomes. Heat maps display the significance of overlap between gene expression signatures comparing aging effects (24M WT/6M WT) versus CD38 deficiency effects (24M CD38KO/24M WT). Color intensity indicates statistical significance (-log10(p-value) of overlapping gene sets. **d,** Expression patterns of the top differentially expressed genes comparing aging (24M WT/6M WT) versus CD38 deficiency effects (24M CD38KO/24M WT) in astrocytes, CA1 neurons, CA3 neurons, and DG. Color scale represents log fold change, with red indicating upregulation and blue indicating downregulation. **e,** Enrichment analysis polar plot of aging hallmarks in genes nominally significant (P <0.01) and discordant between aging and CD38KO in different cell type in the hippocampus; radial length, number of genes; color, −log_10_(*P*-value). **f,** Ingenuity Pathway Analysis (IPA) of CA1 neuron transcriptomics. Bubble plot showing enriched biological pathways, with bubble size representing the number of genes in each pathway and color indicating pathway category. Two key pathways highlighted: “Synaptogenesis Signaling Pathway” and “Glutamate Receptor Signaling Pathway” belong to the most significant signal pathway category. **g,** Detailed heat maps of genes within the synaptogenesis and glutamate receptor signaling pathways. Expression patterns across experimental groups demonstrate that CD38 deficiency enhances expression of synaptic plasticity genes and neurotransmitter signaling components in aged hippocampus.

Given the profound changes in CSF composition observed in CD38KO mice, we next examined whether these alterations translated to metabolic and transcriptional changes in hippocampal tissue, a brain region critical for learning and memory (Fig. 5a). Targeted metabolomic analysis of hippocampal tissue revealed significant enhancement of NAD^+^ metabolism in CD38KO mice (Fig. 5b). NAD^+^ levels were substantially elevated in CD38KO hippocampus, accompanied by increased NMN levels. Conversely, the CD38 enzymatic product NAM was significantly reduced (Fig. 5b). To characterize hippocampal transcriptional states, we performed single-nucleus RNA sequencing (snRNA-seq) of hippocampi from young and aged WT and age-matched CD38KO mice. After quality-control filtering, we analyzed over 42,000 nuclei, identifying all major hippocampal cell types (**Supplementary Fig. 3, Supplementary Table 9,10**), based on canonical marker expression. Pseudo-bulk analysis of the CD38 signature and aging signature of the hippocampus revealed striking reversal of the aging signature in CD38KO hippocampus (Fig. 5c). The analysis showed significant overlap of genes in discordance between the CD38KO and aging signatures, indicating that genes upregulated during aging were suppressed by CD38 deficiency, while genes downregulated during aging were restored. Genes that were significantly upregulated during normal aging showed marked suppression in aged CD38KO mice, returning to levels similar to those of young WT (Fig. 5d). Conversely, genes downregulated with age were restored in CD38KO mice. This reversal pattern was evident across all major cell types (e.g., astrocytes, CA1 neurons, CA3 neurons, and DG neurons) with each showing restoration of youthful gene expression profiles. Enrichment of the nominally significant (P-value <0.01) and discordant gene set between CD38KO and aging (Fig. 5e) within the 12 hallmarks of aging revealed cell type–specific patterns of rejuvenation. Astrocytes and CA3 neurons showed particularly strongly enriched “Altered Intercellular Communication”, while CA1 neurons displayed prominent enrichment with “Disabled Autophagy”, and in DG neurons, “Epigenetic Alteration” was strongly noted. To probe pathway-level changes, we applied Ingenuity Pathway Analysis (IPA) to CA1 neurons. In CA1 neurons, the pathways “Synaptogenesis Signaling” and “Glutamate Receptor Signaling” from the “Neurotransmitters and other Nervous System Signaling” category are most significantly enriched (Fig. 5f). These pathways coordinate upregulation of synaptic structural proteins, neurotransmitter receptors, and downstream signaling molecules in CD38KO CA1 neurons (Fig. 5g). The activation of the synaptogenesis pathway increases synaptic vesicle proteins, cell adhesion molecules, and scaffolding proteins and activation of the glutamate signaling pathway increases expression of AMPA and NMDA receptor subunits and their associated signaling components. Despite low hippocampal expression, CD38 may exert significant effects on hippocampal aging via its elevated levels in the choroid plexus, a key interface regulating cerebrospinal fluid signaling and neuroimmune communication across brain regions.

Thus, these findings suggest that CD38 deficiency in the choroid plexus creates a cascade of beneficial effects extending to hippocampal tissue. The enhanced NAD^+^ availability, combined with transcriptional reprogramming of genes that regulate synapses and reduce age-associated inflammatory signatures, provides a molecular basis for the improved cognitive performance observed in aged CD38KO mice.

### Pharmacological CD38 inhibition with NTX-748 demonstrates brain penetration and target engagement

**Figure 6:**
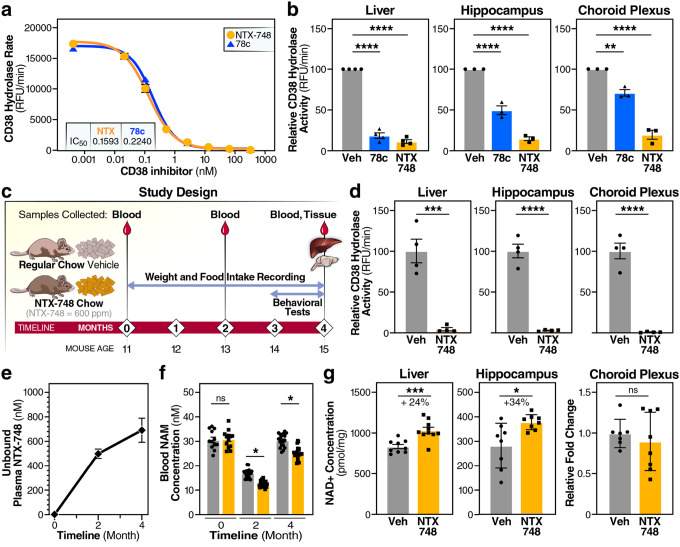
NTX-748 demonstrated potent CD38 inhibition with superior brain penetration and sustained systemic and central NAD^+^ elevation. **a,** *In vitro* CD38 enzymatic inhibition curves showing dose-response relationships for NTX-748 and reference compound 78c using mouse recombinant CD38 protein. Both compounds had potent CD38 inhibition with IC_50_ values of 0.16 nM (NTX-748) and 0.22 nM (78c), showing comparable *in vitro* potency. **b,** *Ex vivo* CD38 hydrolase activity in liver, hippocampus, and choroid plexus tissues harvested 3 hours after oral administration of vehicle, 78c, or NTX-748 at 30 mg/kg. Data represent mean ± s.e.m., **P<0.01, ****P<0.0001 by one-way ANOVA. **c,** Schematic representation of NTX-748 dietary supplementation study design showing experimental timeline, including blood collection, tissue collection, and behavioral testing phases over the course of the study in aging mice. **d,** *Ex vivo* CD38 hydrolase activity in liver, hippocampus, and choroid plexus tissues after chronic dietary supplementation with vehicle or NTX-748. NTX-748 treatment effectively inhibited CD38 activity across all tissues examined, with particularly robust inhibition in brain regions. Data represent mean ± s.e.m., ***P<0.001, ****P<0.0001 by unpaired t-test. **e,** Pharmacokinetic analysis showing unbound plasma concentration of NTX-748 following dietary supplementation (600 ppm) in control diet and NTX-748 diet groups. Measurements taken at months 0, 2, 4 demonstrate sustained drug exposure throughout the treatment period. Data represent mean ± s.e.m., *P<0.05 by one-way ANOVA. **f,** Pharmacodynamic analysis showing blood concentration of NAM following dietary supplementation (600 ppm) in control diet and NTX-748 diet groups. Measurements taken at months 2, 4 demonstrate NAM levels in the NTX-748 treatment were significantly lower in the vehicle control. Data represent mean ± s.e.m., *P<0.05 by unpaired t-test. **g,** NAD+ metabolite analysis in the liver, hippocampus, and choroid plexus from vehicle and NTX-748-treated mice following 4 months of dietary supplementation. Data represent mean ± s.e.m., *P<0.05, ***P<0.001, ns, not significant by unpaired t-test.

To translate our findings into a potential therapeutic approach for cognitive decline during aging, we evaluated NTX-748, a novel brain-penetrant small molecule CD38 inhibitor (**Supplementary Fig. 4**). *In vitro* characterization using recombinant mouse CD38 enzyme showed that NTX-748 had comparable potency to the reference compound 78c^46^ (Fig. 6a). Both compounds exhibited dose-dependent inhibition of CD38 activity with calculated IC_50_ values in the low nanomolar range. Further, NTX-748 demonstrated superior brain penetration and potency compared to 78c. *Ex vivo* tissue analysis after oral administration revealed comparable CD38 inhibition in peripheral tissues such as liver; however, NTX-748 showed markedly greater inhibition of CD38 in the hippocampus and choroid plexus when compared to 78c (Fig. 6b). In the liver, both compounds achieved robust enzyme inhibition, consistent with previous studies showing good peripheral bioavailability of CD38 inhibitors^47^.

To assess long-term efficacy and safety, we conducted a chronic dietary supplementation study in 11-month-old mice(Fig. 6c). Mice received either regular chow or chow supplemented with NTX-748 for 4 months. Blood samples were collected at multiple timepoints to monitor pharmacokinetics, and tissues were harvested at the study endpoint for biochemical analyses (see schematic, Fig. 6c). To evaluate target engagement after chronic NTX-748 treatment, we assessed NAD^+^ hydrolase activity in tissues collected at the study endpoint. NTX-748 treatment resulted in robust inhibition of NAD^+^ hydrolase across all examined tissues. In the liver, hippocampus, and choroid plexus, CD38 activity was nearly completely abolished in NTX-748-treated mice, compared to vehicle controls, confirming effective target engagement in both peripheral and central nervous system tissues (Fig. 6d).

Pharmacokinetic analysis revealed stable drug exposure and pharmacology throughout the treatment period. Unbound plasma concentrations of NTX-748 remained well above the *in vitro* IC_50_ at both 2 and 4 months (Fig. 6e). At the same time points, whole-blood MS analysis revealed markedly lower NAM levels in the NTX-748 group compared with vehicle, consistent with target engagement (Fig. 6f). These results demonstrate that dietary supplementation provides continuous and effective CD38 inhibition.

The sustained CD38 inhibition modulated NAD^+^ metabolism (Fig. 6g). In liver tissue, NTX-748 treatment significantly increased NAD^+^ levels, and the CD38 products NAM and ADPR were reduced (Fig. 6g, **Supplementary Fig. 5**). The magnitude of these changes confirms effective and sustained CD38 inhibition *in vivo*, similar to effects observed with genetic CD38 deletion. NMN levels showed a trend toward an increase, consistent with reduced consumption of this NAD^+^ precursor (**Supplementary Fig. 5**). Similar metabolic changes were observed in brain tissues, indicating that NTX-748 modulates NAD^+^ metabolism in the central nervous system, a critical requirement for neuroprotective effects. Interestingly, NAD^+^ levels in the choroid plexus showed no significant change. This likely reflects tissueand compartment-specific NAD^+^ homeostasis in the choroid plexus. The choroid plexus may act mainly as a gatekeeper; its own NAD^+^ steady state can be tightly buffered and not the rate-limiting step for behavioral outcomes.

### NTX-748 treatment improved cognitive performance in mice

**Figure 7:**
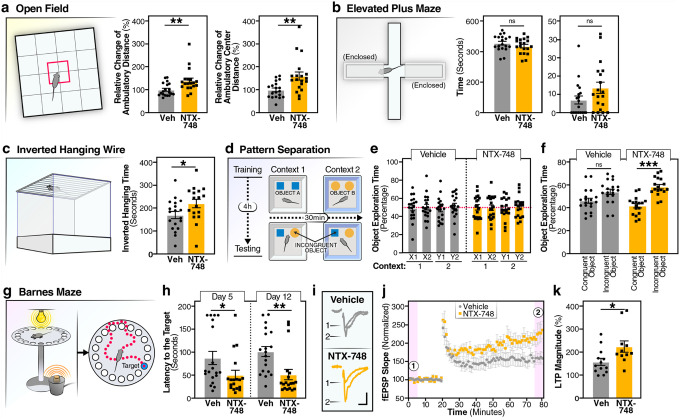
NTX-748 treatment enhanced cognitive performance and synaptic plasticity in aged mice. **a,**Open-field test analysis showing increased ambulatory total distance and ambulatory center distance in NTX-748-treated aged mice, compared to vehicle controls, suggesting enhanced exploratory behavior and reduced anxiety-like behavior. Data represent mean ± s.e.m., **P<0.01 by unpaired t-test. **b,** Elevated-plus maze analysis showing time spent in closed arms versus open arms. Data represent mean ± s.e.m., ns, not significant. **c,**Inverted hanging wire -evaluating muscle endurance and motor function. NTX-748-treated mice had significantly more hanging time than vehicle controls, indicating improved muscle strength and endurance in aged mice after CD38 inhibition treatment. Data represent mean ± s.e.m., *P<0.05 by unpaired t-test. **d-f,** The object context discrimination test of pattern separation memory showing object exploration time for congruent and incongruent objects. NTX-748-treated mice exhibit significantly enhanced discrimination between congruent and incongruent objects, but vehicle-treated mice showed no significant discrimination (ns, not significant). Data represent mean ± s.e.m., ***P<0.001 by paired t-test **g, h,** Barnes maze memory retention testing showing latency to locate the target hole on Day 5 (short-term memory) and Day 12 (long-term memory). Data represent mean ± s.e.m., *P<0.05, **P<0.01 by unpaired t test. **i,** Field excitatory postsynaptic potential (fEPSP) slopes and fiber volley (FV) amplitudes were measured in response to increasing stimulus intensities applied to perforant pathway inputs. Graphs of mean fEPSP slope. **j,** Field excitatory postsynaptic potential (fEPSP) slope measurements showing the 20 minute baseline and 60 minutes after LTP induction. Slopes were normalized to the average fEPSP slope in the 20-minute baseline for each slice. The fEPSP slopes at 55–60 minutes after LTP induction were used to calculate the LTP expression magnitude in **k**(n=11–13 slices / group from 5 mice / group). Data represent mean ± SEM across time. **k,** Quantification of the LTP magnitude showed significantly enhanced expression of LTP in NTX-748-treated mice, compared to vehicle controls, demonstrating improved hippocampal synaptic function after CD38 inhibition (n=11–13 slices / group from 5 mice / group). Data represent mean ± s.e.m., *P<0.05 by unpaired t-test.

To assess the therapeutic efficacy of pharmacological CD38 inhibition, we treated 11-month-old mice with NTX-748 via dietary supplementation and evaluated cognitive and behavioral outcomes (Fig. 6c). In the open field test, a measurement of general locomotor activity and anxiety-related behavior, NTX-748-treated mice showed increased exploratory activity (Fig. 7a). Both total ambulatory distance and, critically, exploration of the anxiogenic center zone were significantly increased in treated mice. The increased center zone exploration suggests reduced anxiety-like behavior, as anxious mice typically avoid open, exposed areas in favor of the protective walls^48^. This anxiolytic effect was further evaluated using the elevated plus maze, where NTX-748-treated mice spent less time in the closed arms and showed a trend toward increased time in the open arms, though the latter did not reach statistical significance (Fig. 7b). Physical function was assessed using the inverted hanging wire test, which evaluates grip strength and muscular endurance—functions that typically decline with age (Fig. 7c). NTX-748-treated mice had better performance with greater hanging time than vehicle controls. This improvement in neuromuscular function suggests that the benefits of CD38 inhibition extend beyond cognitive domains to include preservation of physical capabilities important for healthspan^49^.

Cognitive assessments revealed robust enhancement across multiple memory domains. In the object context discrimination task, which depends on adult hippocampal neurogenesis^50^, NTX-748-treated mice showed dramatically improved performance (Fig. 7d–f). While vehicle-treated mice exhibited no significant discrimination between congruent and incongruent object-context pairings, NTX-748-treated mice demonstrated robust pattern separation memory. This enhancement in pattern separation memory is particularly significant as this cognitive function shows early decline in aging and is sensitive to hippocampal dysfunction^51^. The Barnes maze provided strong evidence for enhanced spatial learning and memory (Fig. 7g,h). Memory retention tests conducted at both short- (day 5) and long-term (day 12) intervals post-training revealed that NTX-748-treated mice showed significantly improved performance, with shorter latencies to locate the target hole at both time points. The improved performance at both intervals indicates that NTX-748 enhances both memory consolidation and maintenance processes. Analysis of search strategies revealed that NTX-748-treated mice predominantly used spatial navigation strategies, whereas vehicle-treated mice relied more on serial searching, indicating qualitatively improved hippocampus-dependent spatial memory formation.

We next evaluated the effect of NTX-748 on long-term potentiation (LTP) in hippocampus, because LTP is widely considered the cellular basis of memory formation^52–54^. To assess LTP in the hippocampus, electrophysiological recordings were performed at Schaffer collateral-CA1 synapses in acute mouse brain slices (Fig. 7i–k, **Supplementary Fig. 6**). We recorded field excitatory postsynaptic potentials in response to stimulation of the Schaffer collateral inputs, and LTP was induced by high frequency stimulation. The LTP magnitude was significantly increased in mice that received NTX-748 treatment compared to vehicle controls (Fig. 7k). This LTP enhancement suggests that CD38 inhibition alters neuronal physiology by promoting synaptic plasticity mechanisms that support improved memory performance in behavioral tests.

## DISCUSSION

We found that age-related CD38 upregulation specifically in choroid plexus pericytes drives tissue dysfunction through NAD^+^ depletion, mitochondrial impairment, and induction of cellular senescence in neighboring epithelial cells. Using genetic deletion in aged mice, we showed that CD38 deficiency preserves choroid plexus metabolic function, reduces epithelial cell senescence, and enriches CSF with beneficial metabolites and proteins. Pharmacological inhibition with the novel CD38 inhibitor NTX-748 recapitulated the metabolic and cognitive benefits, enhancing NAD^+^ levels and improving performance. Our findings establish the choroid plexus as a metabolic gatekeeper of brain aging and position CD38 inhibition as a promising therapeutic strategy for maintaining the rejuvenating properties of CSF and promoting cognitive resilience during aging^55^.

The choroid plexus has emerged as a key interface between systemic aging and brain function^55^. Our findings establish CD38 as a critical regulator of brain aging that operates through an unexpected mechanism: modulation of choroid plexus function via pericytes rather than direct neuronal effects. The predominant expression of CD38 in choroid plexus pericytes, coupled with the improved cognition in CD38 KO mice, reveals a previously unrecognized axis linking peripheral NAD^+^ metabolism to brain health. Conversely, we show age-related CD38 upregulation in choroid plexus pericytes triggered a cascade of deleterious changes: NAD^+^ depletion, mitochondrial dysfunction, epithelial cell senescence, and HMGB1 secretion. These local changes fundamentally altered CSF composition, reducing the metabolic and trophic support provided to brain tissue. The reversal of these changes in CD38-deficient mice—with preserved NAD^+^ levels, reduced senescence, and enhanced CSF quality—provides compelling evidence that the choroid plexus serves as a metabolic gatekeeper for brain aging.

The pericyte-specific expression of CD38 is particularly intriguing. Pericytes regulate blood-brain barrier integrity and cerebral blood flow^56^, but their role in choroid plexus function has been understudied. Our findings suggest that pericyte metabolic health directly influences epithelial cell function through paracrine signaling, potentially involving NAD^+^-dependent pathways, such as sirtuins or PARPs^57^. This intercellular communication paradigm has been observed in other neurodegenerative contexts, where proteostasis decline and endoplasmic reticulum stress in one cell type propagates dysfunction to neighboring cells^58,59^. The prevention of epithelial senescence in CD38KO mice despite unchanged CD38 expression in epithelial cells themselves supports this intercellular communication model. This raises the possibility that pericyte-targeted therapies could indirectly preserve epithelial function and CSF production capacity during aging.

With the high expression of CD38 in the choroid plexus, the comprehensive reversal of hippocampal aging signatures in CD38KO mice suggests that improving choroid plexus function may have far-reaching effects on brain tissue. The uniform benefits across diverse cell types—neurons, astrocytes, and microglia—suggest that enhanced CSF composition may have broad metabolic and trophic support rather than cell-specific effects. This is consistent with recent evidence that CSF-derived factors regulate neurogenesis, synaptic plasticity, and neuroinflammation^27,60,61^. The elevation of NAD^+^ in CSF of CD38KO mice is particularly significant, as NAD^+^ serves not only as a metabolic cofactor but also as a substrate for enzymes regulating DNA repair, gene expression, and stress resistance^62^.

The translation of these findings to pharmacological intervention with NTX-748 has important therapeutic implications. The superior brain penetration of NTX-748 compared to previously reported CD38 inhibitors addresses a key limitation in targeting CNS CD38^46^. The broad behavioral improvements—spanning anxiety, physical function, and multiple cognitive domains—suggest that CD38 inhibition could address several aspects of aging simultaneously. The enhancement of pattern separation is particularly relevant, as this function shows early decline in human aging and correlates with quality of life^51^.

Our findings align with recent studies highlighting the importance of metabolic interventions in aging. The use of NAD^+^ precursors, such as nicotinamide riboside and nicotinamide mononucleotide, has shown promise in preclinical models^63,64^. However, CD38 inhibition may offer advantages by addressing the root cause of NAD^+^ decline rather than simply supplementing precursors. Recent clinical trials have begun to explore NAD^+^ augmentation strategies in humans^65^, and our work suggests that CD38 inhibition could be a particularly effective approach. Beyond preserving NAD^+^, targeting CD38 may confer benefits by reshaping intracellular Ca2^+^ signaling. Through its generation of cADPR and ADPR, CD38 modulates Ca2^+^ homeostasis via ryanodine receptors and TRPM2 channels, potentially affecting mitochondrial function, barrier properties, and secretory programs in choroid plexus epithelium and pericytes. Thus, some favorable phenotypes with CD38 inhibition could reflect Ca2^+^-dependent mechanisms rather than NAD^+^ preservation alone. Future studies that selectively perturb cADPR/ADPR pathways will help disentangle Ca2^+^-mediated effects from NAD^+^-dependent benefits.

Several aspects of our study warrant discussion. First, our studies were conducted exclusively in mice, and the translational relevance to human aging remains to be established. While CD38 expression increases with age in human tissues^66^, the specific role of choroid plexus CD38 in human cognitive aging is unknown. Species differences in choroid plexus anatomy and function could influence therapeutic efficacy. Second, the long-term safety of CD38 inhibition requires careful evaluation. CD38 has important roles in immune function and calcium signaling^67,68^ and chronic inhibition could have unintended consequences, particularly in the context of infection or injury. Finally, our study centers on pericyte-epithelial interactions and does not comprehensively profile the immune compartment of the aging choroid plexus. Recent reports implicate macrophage-derived cathepsin S in blood-CSF barrier disruption^66^, but we did not assess immune regulation here.

Importantly, neither our genetic nor pharmacological approaches provide brain-specific targeting, which represents a significant limitation in interpreting our results. The CD38KO is a whole-body knockout mouse, affecting all tissues including peripheral immune cells, liver, and muscle. Similarly, NTX-748 is administered systemically and achieves CD38 inhibition throughout the body. The cognitive improvements we observed could partially or even predominantly result from peripheral effects such as improved systemic metabolism, reduced peripheral inflammation, or enhanced muscle function—all of which can indirectly influence brain health. Definitive proof that the choroid plexus-CSF-brain axis is the primary mechanism will require more targeted approaches.

Regarding safety, long-term administration of the CD38 inhibitor 78c has been shown to be not only safe but also to extend lifespan and healthspan in mice^17^, providing encouraging preliminary evidence for the feasibility of chronic CD38 inhibition. Nevertheless, CD38’s roles in immune function, particularly in T cell activation, antibody responses, and neutrophil chemotaxis^69^, warrant careful consideration. Chronic CD38 inhibition could potentially compromise immune surveillance or response to infections, particularly concerning in elderly populations who already face increased susceptibility to infections. The timing, duration, and degree of CD38 inhibition may be critical factors in balancing therapeutic efficacy with potential adverse effects.

The broader implications of our findings extend beyond CD38 to the general strategy of targeting the choroid plexus for brain rejuvenation. Young CSF can improve brain function in aged mice^60^, and our work provides a mechanistic framework for these observations. The choroid plexus may represent a more accessible therapeutic target than neurons themselves, as systemically administered drugs can more readily reach this highly vascularized structure. Combination therapies targeting multiple aspects of choroid plexus aging (e.g., senescence, inflammation, and metabolic dysfunction) might provide synergistic benefits. The choroid plexus-CSF-brain axis as a key regulator of cognitive aging may also have diagnostic implications. The development of imaging techniques to assess choroid plexus function could enable non-invasive monitoring of brain health and therapeutic efficacy^70^. Future directions should include investigation of CD38 expression patterns in human choroid plexus across the lifespan, assessment of CSF NAD^+^ levels in aging and cognitive impairment, and development of additional brain-penetrant CD38 inhibitors with improved pharmacological properties. The potential for CD38 inhibition to synergize with other anti-aging interventions, such as senolytics or NAD^+^ precursors, should also be explored.

In conclusion, our study reveals CD38-mediated choroid plexus dysfunction as a targetable driver of cognitive aging. The behavioral and electrophysiological data show that pharmacological CD38 inhibition with NTX-748 robustly improves brain function in aging. NTX-748 reduced anxiety-like behavior, enhanced neuromuscular performance, and strengthened hippocampal synaptic plasticity, as evidenced by increased LTP magnitude. Cognitively, NTX-748 improved pattern separation and shortened latencies in spatial memory testing, indicating benefits across multiple memory domains. The LTP enhancement provides a mechanistic link between improved choroid plexus–CSF milieu and hippocampal circuit performance, supporting NTX-748 as a promising therapeutic approach to bolster cognitive resilience during aging.

## METHODS

### Animals and experimental design

All animal studies were conducted in accordance with protocols approved by the Institutional Animal Care and Use Committee at the Buck Institute for Research on Aging (protocol no. A10285). Male C57BL/6J wild-type mice and CD38 knockout (CD38 KO) mice on a C57BL/6J background (Jackson Laboratory) were used. Animals were housed in a specific pathogen-free facility under a 12 h light/12 h dark cycle, at an ambient temperature of 20–23°C, and 30–70% relative humidity. Age-matched mice were randomly assigned to experimental groups. Unless otherwise noted, animals were maintained on a standard chow diet (Teklad Global 18% Protein Rodent Diet, Envigo, cat. no. 2018). Behavioral testing was conducted at specified time points as described in the following sections.

### Drug treatment

NTX-748, a small molecule CD38 inhibitor, was kindly provided by our collaborators at Napa Therapeutics Ltd (a Juvenescence Ltd. company). For short-term *ex vivo* studies, mice received a single oral gavage of NTX-748 (30 mg/kg body weight) or vehicle control. Tissues were collected 3 h post-administration for the hydrolase activity assays. For the long-term dietary intervention, mice were fed either a control diet (Irradiated Global 14% Protein Diet 2914, Inotiv-Teklad Diets) or the same diet supplemented with NTX748 (600 ppm). Diets were provided ad libitum for a period of 4 months.

### Behavioral assessments

#### Open field.

Open field was performed using the TRU SCAN Activity Monitoring System (Coulbourn Instruments). Mice were habituated for 30 min in the test room with lights off before testing. Mice were gently placed in a corner of the arena and allowed to move freely for 15 min while being monitored by the automated photobeam tracking system. TruScan’s precision is 10 inch × 10 inch, twice the cage’s beam resolution; 0.5 inch (1.27 cm) for the beam spacing of the large cage and 0.3 inch (0.76 cm) for spacing of the small cage. The total sum of elapsed time of all movements in the floor plane and the total sum of all vectored coordinate changes (move distance) in the floor plane were calculated. Stereotypic movement is defined as the total number of coordinate changes less than ±0.999 beam spaces in each floor plane (X and Y) dimension and back to the original point that do not exceed 2 s apart. Movements that are less stereotypic were calculated as ambulatory movements. The arena center is the region that is more than 2.5 beam spaces away from the arena walls. Parameters measured included total distance traveled, ambulatory distance, center distance, margin distance, center time, and number of center entries.

#### Barnes maze.

Spatial learning and memory were assessed using the Barnes maze. The circular platform (120-cm diameter) contained 40 holes around the perimeter, with one target hole containing an escape box. Mice underwent 4 consecutive days of training with two trials per day and 4-h inter-trial intervals. During both training and testing, bright light and loud white noise were applied as aversive stimuli until mice entered the escape box (during training) or found the target location (during probe tests). Latency to locate the target hole was recorded during training with a maximum trial duration of 180 s. Probe tests were conducted on days 5 and 12 by removing the escape box (goal box). During probe tests, the cumulative duration when both the center point and nose point were in the same goal box entry zone for more than 3.0 s was considered as finding the target. Both training and testing sessions were recorded for 180 s and analyzed using EthoVision XT 15 software.

#### Elevated plus maze.

Anxiety-like behavior was assessed using the elevated plus maze. The apparatus consisted of two open arms (36 × 8 cm) and two closed arms (36 × 8 cm) extending from a central platform (8 × 8 cm), elevated 80 cm above the floor. Mice were placed on the central platform facing an open arm and allowed to explore freely for 10 min under dim lighting conditions. Parameters measured included time spent in open arms, number of open arm entries, total arm entries, and percentage of open arm entries. All sessions were recorded and analyzed using EthoVision XT 15 software. Increased time and entries in open arms indicate reduced anxiety-like behavior.

#### Inverted hanging grip.

Motor coordination and muscle strength were evaluated using the inverted hanging grip test. Mice were placed on a wire mesh grid (40 × 60 cm with 1-cm^2^ openings) that was then inverted 50 cm above a soft surface. The latency to fall from the inverted grid was recorded with a maximum test duration of 600 s.

#### Object context discrimination.

Pattern separation memory was assessed using an object context discrimination task. The test consisted of two distinct contexts: Context 1 (white walls, cleaned with 1% acetic acid) and Context 2 (checkered pattern, cleaned with 70% ethanol). During the training phase, mice explored two identical objects (Object A in Context 1, Object B in Context 2) for 10 min in each context with a 30-min interval between contexts. After a 4-h retention interval, mice were tested in both contexts. In Context 1, one Object A was replaced with one Object B (incongruent object from Context 2). In Context 2, one Object B was replaced with one Object A (incongruent object from Context 1). Pattern separation memory was measured by calculating the discrimination ratio for the incongruent object in each context: (time exploring incongruent object - time exploring congruent object)/(total exploration time). Higher discrimination ratios indicate better pattern separation memory.

### Tissue and plasma collection and processing

Mice were anesthetized with ketamine/xylazine (80 mg/kg and 10 mg/kg, respectively with IP injection). Blood was collected via cardiac puncture using EDTA-coated syringes after anesthesia induction. Whole blood was centrifuged at 2,000 × g for 15 minutes at 4°C, and plasma was carefully collected from the supernatant, and snap-frozen in liquid nitrogen. After CSF (see below) and blood collection, mice underwent transcardial perfusion with PBS or were processed without perfusion, depending on the downstream applications. Brains were rapidly removed and dissected on ice. Hippocampi and choroid plexus tissues were isolated under a dissecting microscope, snap-frozen in liquid nitrogen, and stored at −80°C until analysis. For histological analyses, mice were transcardially perfused with 4% paraformaldehyde in phosphate-buffered saline.

### Cerebrospinal fluid collection

CSF was collected according to the STAR protocol^71^ with modifications. Mice were anesthetized with ketamine/xylazine (80 mg/kg and 10 mg/kg, respectively with IP injection) and positioned prone on a stereotactic frame with the head flexed downward. Glass capillaries were prepared by pulling glass tubes using a micropipette puller, then trimmed to create a flat end with an appropriate diameter. The capillary was positioned horizontally and connected to an aspirator tube. CSF was extracted by puncturing the cisterna magna with the glass capillary positioned parallel to the mouse’s nose. CSF collection was completed within 4–5 min per mouse, typically yielding 8–15 μL per mouse. Samples were immediately examined under a magnifying glass to check for blood contamination, and only clear, uncontaminated CSF samples were used for subsequent analyses. Quality control for blood contamination was performed using a NanoDrop spectrophotometer at 415 nm wavelength, the most sensitive wavelength for detecting oxyhemoglobin, with samples showing absorbance >0.023 AU being excluded from analysis based on the clinical cutoff for intracranial hemorrhage. CSF was dispensed into pre-chilled microcentrifuge tubes, centrifuged at 1,000g for 10 min at 4°C to remove any cellular debris, and stored at −80°C until analysis. This is a terminal procedure, and mice were euthanized immediately after CSF collection.

### Western blot analysis

Tissue lysates were prepared using T-PER Tissue Protein Extraction Reagent (Invitrogen, 78510) containing protease and phosphatase inhibitors. Protein concentrations were determined using the BCA assay. Equal amounts of protein were separated by SDS-PAGE and transferred to nitrocellulose membranes using a Bio-Rad Trans-Blot Turbo semi-dry transfer system. Membranes were blocked with 5% non-fat milk in TBS-T for 1 h at room temperature (RT) and incubated overnight at 4°C with primary antibodies: CD38 (1:1,000, Abcam ab216343), and GAPDH (1:5,000, Proteintech, 60004-1-1g). After washing with TBS-T, membranes were incubated with HRP-conjugated secondary antibodies (anti-rabbit or anti-mouse, 1:5,000, Cell Signaling Technology, 7074 and 7076, respectively) for 1 h at RT. Proteins were detected using enhanced chemiluminescence substrate and images were captured using Azure C300 and C600 imaging systems. Protein bands were quantified by densitometry using ImageJ software and normalized to GAPDH loading control.

### Immunofluorescence microscopy

Formalin-fixed paraffin-embedded (FFPE) blocks were sectioned coronally at 5 μm thickness containing lateral and third ventricles with choroid plexus. FFPE whole brain sections (5–15 μm) were deparaffinized in xylene (2 × 5 minutes) and rehydrated through graded ethanol series (100%, 95%, 95%, 70%, 50%) to distilled water. Antigen retrieval was performed in Tris-EDTA buffer (pH 9.0) for 15 min using a pressure cooker. Sections were blocked with 10% donkey serum and 2% BSA in TBS for 1 h at RT, and incubated overnight at 4°C with primary antibodies: CD38 (1:200, Abcam ab216343; 1:500, R&D Systems AF4947), p21 (1:200, Abcam ab188224), HMGB1 (1:200, Cell Signaling 6893), TTR (1:500, Abcam ab215202; 1:200, Thermo Fisher Scientific 66108-1-1G), CD13 (1:200, Cell Signaling 32720; 1:200, Proteintech 66211-1-1g), CD31 (1:50, Dianova DIA-310), PDGFR (1:200, Abcam ab32570), and E-Cadherin (1:200, Cell Signaling 14472). After three washes with TBS, sections were incubated with species-appropriate Alexa Fluor secondary antibodies (1:500, Invitrogen, donkey anti-rabbit, 488nm, A21206; donkey anti-rabbit, 594nm, A31573; donkey anti-rabbit, 647nm, A31573; donkey anti-mouse, 488nm, A21202; donkey anti-mouse, 594nm, A32744; donkey anti-mouse, 647nm, A31571; donkey anti-sheep, 594 nm, A11016; donkey anti-rat, 488nm, A21208; donkey anti-rat, 647nm, A78947) for 1 h at RT in the dark and counterstained with DAPI (5 μg/mL, Invitrogen D1306). Images were acquired using a Keyence BZ-X810 fluorescence microscope for overview imaging and a Zeiss LSM 980 confocal microscope with appropriate laser lines and emission filters for high-resolution analysis. Quantitative analysis was performed using Image Analyst MKII (Image Analyst Software, Novato CA) using the “AI Fluorescence and absorbance histometry using nuclear and secondary whole cell segmentation (1–4 labels - advanced background options)” with Cellpose^72^ and colocalization analysis using the “Plot Correlation” function.

### GeoMx Digital Spatial Profiling (DSP)

Spatial transcriptomic profiling of the choroid plexus was performed on formalin-fixed, paraffin-embedded (FFPE) brain sections obtained from CD38 wild-type (WT) and knockout (KO) mice at 6 and 24 months of age. Brains were fixed in 10% neutral-buffered formalin for 24 h, paraffin-embedded, and sectioned coronally at 5 μm thickness to include the lateral and third ventricles. Sections were mounted on Superfrost Plus slides (Thermo Fisher Scientific) and processed for spatial transcriptomics according to the NanoString GeoMx DSP Slide Preparation User Guide (MAN-10150–05, updated November 2023)^73^. Slides were baked at 60 for 2 h, deparaffinized in xylene (3 × 5 min), and rehydrated through graded ethanol (2 × 5 min in 100% EtOH, 1 × 5 min in 95% EtOH), followed by a rinse in phosphate-buffered saline (PBS). Antigen retrieval was performed in 10 mM Tris 1 mM EDTA buffer (pH 9.0) using a laboratory steamer at 100°C for 15 min. Sections were then permeabilized with proteinase K (0.1 mg/mL) for 15 min at 37°C and washed in PBS. Hybridization was carried out overnight at 37°C using 250 μL of GeoMx probe mix (25 μL mouse Whole Transcriptome Atlas probes, 200 μL Buffer R, 25 μL nuclease-free water; NanoString Technologies) under HybriSlip coverslips (Grace Bio-Labs). Coverslips were removed by immersion in 2XSSC containing 0.1% (v/v) Tween-20, followed by two stringent washes (25 min each, 50% formamide in 2XSSC at 37°C) and a final rinse in 2XSSC for 5 min. Sections were blocked for 1 h at RT in Buffer R supplemented with 7% (v/v) donkey serum. Nuclei were counterstained with Syto 83 (Thermo Fisher Scientific; 10 min, RT), rinsed in PBS, and loaded onto the GeoMx Digital Spatial Profiler (NanoString Technologies) for region-of-interest (ROI) selection and oligonucleotide collection. ROIs were defined in GeoMx DSP Software v2.0, with photocleaved oligonucleotide tags aspirated from each ROI into individual wells of a 96-well PCR plate. GeoMx Digital Spatial Profiling was performed on brain sections from 4 mice per experimental group, with 3 biological replicate sections analyzed per mouse. For each section, 3–5 ROIs were selected, encompassing both the lateral ventricle and third ventricle areas (**Supplementary Table 1**). Oligonucleotide eluates were dried and resuspended in 10 μL of DEPC-treated nuclease-free water; 4 μL from each eluate served as template for PCR library construction using the GeoMx SeqCode Dual-Index Primer Mix (NanoString Technologies; MAN-10153–01). Libraries were sequenced on an Illumina NovaSeq 6000 platform, achieving >98% sequencing saturation at a target depth of approximately 1,000 reads per probe per ROI.

Geomx DCC files were input for analysis using readNanoStringGeoMxSet from the GeomxTools package (doi:10.18129/B9.bioc.GeomxTools). Sample information was inputed into a spatial experiment file for conversion to a DGEList object compatible with edgeR using geomxNorm and spe2dge functions from the standR package (PMC10783521). The edgeR package was used to estimate dispersions for 110 samples (ROIs) using the sample ID and choroid plexus regions (left, middle or right choroid plexus: ~ID + region) and fit with glmFit using the group (old/young and wt/ko), region and slide number (~0+group+slide+region). Non-log normalized counts from the cpm function of edgeR and residuals (type=”deviance”) were used as input for RUVr function from the RUVSeq package^74^ with k=17. Differential gene expression was calculated using the previous design matrix and including the seventeen factors of unwanted variation from RUVr (~0+group+W_1+W_2+W_3+W_4+W_5+ W_6+W_7+W_8+W_ 9+W_10+W_11+W_12+W_13+W_14+W_15+W_16+W_17). Samples were fit with glmQLFit and contrasts between old WT/young WT and old CD38KO/old WT were calculated with glmQLFTest. Enrichment, DEG, RRHO analysis was performed as described below. The raw counts and DEGs are in **Supplementary Table 2**.

### Mass spectrometry-based metabolomics

#### Targeted Metabolomics

##### Sample Preparation

Extraction of NAD^+^ and related metabolites from liver, choroid plexus (lateral and the 3rd ventricle) and hippocampus was performed as previously described^75,76^ with minor modifications. Briefly, frozen pre-weighed tissue samples (~15–20 mg) were homogenized in 80% methanol containing an internal standard mix (13C–NAD^+^ and 13C–NAM) using a bullet blender (Next Advance bead homogenizer) for two cycles (speed 8, 2 min each). Homogenates were vortexed for 10 s, incubated on ice for 30 min, and centrifuged at 18,000 rcf for 10 min. The resulting supernatant was dried under vacuum and stored at −20°C until resuspension in the initial LC buffer for analysis. A mixed calibration stock solution (1 mM) of NAD^+^, NAM, ADPR, and NMN was prepared in HPLC-grade water. Calibration curves were generated by serial 2.5-fold dilutions of the stock, ranging from 1000 μM to 0.065 μM. Protein estimation was determined from the protein lysate for choroid plexus samples.

##### LC–MS Analysis

Metabolomics data were acquired using a Q Exactive mass spectrometer coupled to a Vanquish UHPLC system (Thermo Fisher Scientific). Separation of NAD^+^ metabolites were achieved on a Hypercarb column (5 μm, 100 × 2.1 mm; Thermo Fisher Scientific) maintained at 60°C. The mobile phases consisted of (A) 7.5 mM ammonium acetate with 0.05% (v/v) ammonium hydroxide in water, and (B) 0.05% (v/v) ammonium hydroxide in acetonitrile. The gradient was run at 0.25 mL/min over 12 min as follows: 0–1 min, 5% B; 1–6 min, 5–60% B; 6.1–7.5 min, 90% B; 7.6–12 min, 5% B. The Q Exactive MS source parameters, optimized by direct infusion of NAD^+^ metabolites, were: sheath gas flow 45, auxiliary gas flow 2, spray voltage 3.75 kV, capillary temperature 320 °C, S-lens RF 55, and probe temperature 120°C. Data were acquired in positive ion mode using Full MS/ddMS2 (Top 4) and targeted single/parallel reaction monitoring (tSIM–PRM) within a scan range of m/z 50–750. Full MS scans were collected at 70,000 resolution (AGC target 1e6; max IT 100 ms). MS2 scans were acquired at 17,500 resolution (AGC target 1e5; max IT 50 ms; 1.2 m/z isolation window; stepped collision energy 10, 20, 40). For targeted acquisition, an inclusion list of NAD^+^ metabolites was scheduled with a loop count of 6. Normalized collision energies for individual metabolites were optimized by direct infusion.

##### Data Processing and Quantification

Metabolites were identified by matching retention times and MS1/MS2 spectra against authentic standards, and further validated using online databases (Metlin, MassBank). Characteristic fragments of NAD^+^ metabolites were confirmed. Extracted ion chromatograms were used to calculate the normalized area under the curve (AUC). Relative metabolite levels were quantified by comparing analyte/internal standard ratios (13C–NAD^+^, 13C–NAM). Absolute concentrations were determined using a 8-point calibration curve. Data were processed with Thermo Freestyle and Xcalibur Quan Browser software. Quality assurance included blanks, pooled QC samples, and calibration standards to monitor matrix effects and instrument drift. Data from liver and hippocampus were normalized to their corresponding tissue weights while choroid plexus data was normalized to the corresponding total protein in each sample.

### Proteomics analysis.

#### Protein Digestion and Desalting

Crude CSF and plasma samples were prepared as follows: crude CSF and plasma samples (2 μL) were brought to the same overall volume of 50 μL with water. All samples were reduced using 20 mM dithiothreitol in 50 mM TEAB at 50°C for 10 min, cooled to room RT and held at RT for 10 min, and alkylated using 40 mM iodoacetamide in 50 mM TEAB at RT in the dark for 30 min. Samples were acidified with 12% phosphoric acid to obtain a final concentration of 1.2% phosphoric acid. S-Trap buffer consisting of 90% methanol in 100 mM TEAB at pH ~7.1, was added and samples were loaded onto the S-Trap micro (biofluids) spin columns (Protifi). The entire sample volume was spun through the S-Trap micro or mini spin columns at 4,000 × g and RT, binding the proteins to the micro spin columns. Subsequently, S-Trap micro spin columns were washed twice with S-Trap buffer at 4,000 × g and RT and placed into clean elution tubes. Samples were incubated for one-hour at 47°C with sequencing-grade trypsin (Promega, San Luis Obispo, CA) dissolved in 50 mM TEAB at a 1:25 (w/w) enzyme:protein ratio. An additional aliquot of trypsin dissolved in 50 mM TEAB was added and samples were digested overnight at 37°C. Peptides were sequentially eluted from micro S-Trap spin columns with 50 mM TEAB, 0.5% formic acid (FA) in water, and 50% acetonitrile (ACN) in 0.5% FA. After centrifugal evaporation, crude CSF and plasma were desalted using in-house packed C_18_ tips. Desalted elutions were subjected to an additional round of centrifugal evaporation and re-suspended in 20 microliters of 0.2% FA in water. Crude CSF samples were subjected to a 1:2 dilution, whereas crude plasma samples were diluted by a factor of 1:40. 1 μL of indexed Retention Time Standard (iRT, Biognosys, Schlieren, Switzerland) was added to each sample, thus bringing up the total volume to 11 μL (all CSF and plasma samples)^77^.

#### Mass Spectrometric Analysis

Reverse-phase HPLC-MS/MS analyses were performed on a Dionex UltiMate 3000 system coupled online to an Orbitrap Exploris 480 mass spectrometer (Thermo Fisher Scientific, Bremen, Germany). The solvent system consisted of 2% ACN, 0.1% FA in water (solvent A) and 80% ACN, 0.1% FA in ACN (solvent B). Digested peptides (1:2 dilution, crude CSF; 1:40 dilution, crude plasma) were loaded onto an Acclaim PepMap 100 C18 trap column (0.1 × 20 mm, 5 μm particle size; Thermo Fisher Scientific) over 5 min at 5 μL/min with 100% solvent A. Peptides were eluted on an Acclaim PepMap 100 C18 analytical column (75 μm × 50 cm, 3 μm particle size; Thermo Fisher Scientific) at 300 nL/min using the following gradient: linear from 2.5% to 24.5% of solvent B in 125 min, linear from 24.5% to 39.2% of solvent B in 40 min, up to 98% of solvent B in 1 min, and back to 2.5% of solvent B in 1 min. The column was re-equilibrated for 30 min with 2.5% of solvent B, and the total gradient length was 210 min. Each sample was acquired in data-independent acquisition (DIA) mode^78–80^. Full MS spectra were collected at 120,000 resolution (Automatic Gain Control (AGC) target: 3e6 ions, maximum injection time: 60 ms, 350–1,650 m/z), and MS2 spectra at 30,000 resolution (AGC target: 3e6 ions, maximum injection time: Auto, Normalized Collision Energy (NCE): 30, fixed first mass 200 m/z). The isolation scheme consisted of 26 variable windows covering the 350–1,650 m/z range with an overlap of 1 m/z (see **Supplemental Table 3**)^78^.

#### DIA-MS Data Processing and Statistical Analysis

DIA data was processed in Spectronaut (version 19.9.250324.62635) using directDIA. Data extraction parameters were set as dynamic and non-linear iRT calibration with precision iRT was selected. Data was searched against the *Mus musculus* reference proteomes with and 54,739 entries (UniProtKB-SwissProt), accessed on 03/04/2025. Trypsin/P was set as the digestion enzyme, and two missed cleavages were allowed. Cysteine carbamidomethylation was set as a fixed modification while methionine oxidation and protein N-terminus acetylation were set as dynamic modifications. Identification was performed using 1% precursor and protein q-value. Quantification was based on the peak areas of extracted ion chromatograms (XICs) of 3 – 6 MS2 fragment ions, specifically b- and y-ions, with q-value sparse data filtering and iRT profiling applied (**Supplemental Tables 4,5**). Local normalization and differential expression was applied for all samples (**Supplemental Tables 6,7**). Local normalized proteomic values of CSF and plasma from nine paired old wild-type and nine paired old knockout samples were log2 median normalized with the PRONE package^81^. Normalized values were fit with lmFit from the limma package^82^ using the groups (WT/KO and CSF/plasma: ~group). Residuals, calculated with residuals. LArrayLM, and 2^(median normalized counts) were used as input for ruvR with k=6. Differential protein translation was calculated using the previous design matrix and including the five factors of unwanted variation from RUVr (~0+group+W_1+W_2+W_3+W_4+W_5+ W_6). Correlation between samples from the same animal ID were determined with duplicateCorrelation with the block set as the animal ID. Contrasts between old CD38KO/old WT and CSF/plasma were calculated with eBayes and topTable adjusted by FDR (**Supplementary Table 8**). Volcano plots were created using EnhancedVolcano package (doi:10.18129/B9.bioc.EnhancedVolcano). Enrichment was performed as described above in the spatial transcriptomics section.

#### Mitochondrial respiration analysis

Choroid plexus explants were dissected in modified Neurobasal medium supplemented by (5.5 mM glucose, 2 mM L-glutamine, 1 mM pyruvate, 1.8 mM CaCl2, 0.812 mM MgCl2, 89 mM NaCl, 20 mM HEPES, pH 7.4) and maintained on ice. Tissue explants were plated on Cell-Tak-coated (Corning) XFe96 spheroid microplates (Agilent, 102905–100) and equilibrated for 1 hour at 37°C in a non-CO_2_ incubator. Oxygen consumption rates were measured using a Seahorse XFe96 Extracellular Flux Analyzer (Agilent Technologies). Sequential injections of oligomycin (2 μg mL−1), FCCP (2 μM), and rotenone/antimycin A (1 μM each) were performed to determine basal, ATP-linked, maximal, and non-mitochondrial respiration, respectively.

#### NAD^+^ hydrolase activity

Measurement of NADase inhibitory activity of the compounds 78c and NTX-748 was measured using a fluorescence-based assay with ε-NAD^+^ as substrate. Recombinant mouse CD38 (50 ng mL−1) was pre-incubated with varying concentrations of NTX-748 or reference compound 78c (from 5.12 pM to 400 nM) in assay buffer (250 mM sucrose, 40 mM Tris-HCl pH 7.4, supplemented with protease and phosphatase inhibitors). Reactions were initiated by the addition of 80 μM ε-NAD^+^, and fluorescence (λ = 320 nm, λ = 410 nm) was monitored continuously using a CLARIOstar microplate reader (BMG Labtech, Ortenberg, Germany).

Measurement of NAD^+^ hydrolase activity from mouse tissues was performed using a fluorescence-based NADase activity assay. Tissues (liver, hippocampus and choroid plexus) were harvested from C57BL/6 (age is noted in the figure legend, n = 3–4). After collection, tissues were placed in a sucrose buffer (250 mM Sucrose (Sigma Aldrich, 57-50-1), 40 mM Tris pH 7.5 (VWR, 97062-936) with 1X Halt protease and phosphatase inhibitor Cocktail (Thermo Fisher Scientific, 78447 suspended in milliQ water) on ice. Tissue samples were homogenized in 200–300 μL assay buffer using probe sonication (5 cycles of 2 s on/2 s off at 40 W). Homogenates were centrifuged at 15,000×g for 10 min at 4°C and supernatant transferred to a new pre-chilled tube. Protein measurement was performed using the Pierce BCA Protein Assay Kit (Thermo Fisher Scientific, 23225) following the manufacturer’s provided protocol. Then, 10 μg of protein in 100 μL of sucrose buffer per sample was transferred in a 96-well black plate (Corning, CLS3650). 5 μL of Nicotinamide 1,N6-ethenoadenine dinucleotide (Sigma Aldrich, N2630) 0.8 mM was added to reach the final concentration of 80 μM and start the reaction. The CLARIOstar Plus plate reader (BMG Labtech, Ortenberg, Germany) was used to measure fluorescence at 300 nm excitation and 410 nm emission every 60 s for 30 min. Following assay completion, the NADase activity was extrapolated as the slope of the linear portion of the Fluorescence over time curve.

#### Single Nucleus RNA Sequencing (snRNA-seq) Using 10x Genomics Platform

Hippocampal tissues were dissected from young (6-month-old) wild-type (n=5), aged (24-month-old) wild-type (n=3), young (6-month-old) CD38KO (n=3), and aged (24-month-old) CD38KO (n=4) mice processed for nuclei isolation (10X Genomic, 1000494). Frozen hippocampal samples were mechanically homogenized and incubated on ice for 5 minutes, followed by sequential filtration through 40 μm and 30 μm strainers to remove debris and tissue aggregates. Nuclei were pelleted by centrifugation (500 g, 5 min, 4°C), washed twice in nuclei suspension buffer, and assessed for integrity using the Cellaca PLX imaging cytometer with trypan blue viability exclusion to ensure >90% intact nuclei. Following concentration adjustment to 1,000 nuclei/μL, approximately 13,000 nuclei per sample were loaded onto the Chromium Next GEM chip to target ~10,000 captured nuclei per library. Single-nucleus libraries were generated using the automated Chromium Connect instrument with the 10x Genomics Next GEM Single Cell 5’ Kit v2 (10x Genomics, 1000290), which performed GEM generation, barcoding, reverse transcription, cDNA amplification, and dual sample indexing in a walk-away protocol. Library quality was verified using Agilent TapeStation High Sensitivity DNA ScreenTape and quantified via Qubit dsDNA HS assay before sequencing on Illumina NovaSeq 6000 S4 flow cells (28 bp Read 1, 90 bp Read 2) at a median depth of 50,000 reads per nucleus. Raw FASTQ files were processed through Cell Ranger v7.0^83^ aligned to the mm10 (GRCm38) reference genome to generate digital gene expression matrices.

Each sample was normalized and scaled using SCTransform from Seurat (version 5.1). Doublets were identified using DoubletFinder^84^, using the optimal number of principal components (PCs) using findPC (https://doi.org/10.1093/bioinformatics/btac235), assuming 7.5% doublet formation and optimal pK using the same number of PCs. Doublet removed counts were normalized and scaled with SCTransform, PCA with RunPCA, the optimal number of PCs identified with findPC used for RunUMAP and FindNeighbors followed by clustering with FindClusters. Ambient RNA was removed using decontx^85^ with doublet removed counts and the identified clusters. Seurat objects from the doublet removed and ambient RNA corrected samples were merged followed by normalization and scaling using SCTransform. Dimensional reductions using PCA RunPCA and iPCA from the mixOmics package^86^ were calculated for the top 50 PCs. Samples were split by library batch construction and integrated using HarmonyIntegration through IntegrateLayers followed by FindNeighbors and FindClusters. Cell markers (**Supplementary Table 9**) for known cell types were used to classify each cluster. Cell types not present in each sample were removed from further analysis. The remaining 40,920 cells were used in downstream analyses. Final UMAPs were constructed from IPCA constructed from the scaled data after running NormalizeData, FindVariableFeatures and ScaleData on the doublet removed, ambient RNA removed cell counts (**Supplementary Table 10**).

DEG analysis was performed on pseudobulk data by sample and cell type or by sample using AggregateExpression on young/old and WT/CD38KO samples. The edgeR^87^ package was used to estimate dispersions for the four groups (young/old and WT/CD38KO with design matrix: ~group) and fit with glmFit. Non-log normalized counts from the cpm function of edgeR and residuals (type=”deviance”) were used as input for RUVr function from the RUVSeq package^74^ with k=5. Exploratory analyses not including differential gene expression used normalized counts from RUVr. Differential gene expression was calculated for the 15 samples (4 old CD38KO, 3 old WT, 5 young WT and 3 young CD38KO) using the previous design matrix and including the five factors of unwanted variation from RUVr (~0+group+W_1+W_2+W_3+W_4+W_5). Samples were fit with glmQLFit and contrasts between old WT/young WT and old CD38KO/old WT were calculated with glmQLFTest. Rank-rank hypergeometric overlap (RRHO) of ranked gene sets by log fold change were calculated using the RRHO2. Gene module scores were calculated with the escape package^88^ with GSVA and the pseudobulked counts using cell-specific aging gene sets that are discordant in knock out comparisons (old versus young with p-value <0.01 and old knock versus old with log fold change values discordant with old versus young log fold change values) or also significant in knock out comparisons (*P*-value <.01). Gene enrichment using the GeneOverlap package (doi:10.18129/B9.bioc.GeneOverlap) and GSEA from the clusterProfiler package^89^ using gene sets for hallmarks of aging^42^ and senescence-specific genes from CellAge^90^, SenMayo^91^, SASP Atlas (fibroblast and epithelial)^92^, REACTOME^93^ and GO^94^. enrichGO and gseGO from the clusterProfiler package were used for gene enrichment analysis of GO terms.

#### Electrophysiology

Mouse brains were rapidly dissected in cold sucrose solution containing (in mM): 210 sucrose, 2.5 KCl, 1.25 NaH_2_PO_4_, 25 NaHCO_3_, 7 glucose, 2 MgSO_4_, and 0.5 CaCl_2_ (perfused with 95% O_2_, 5% CO_2_ with pH ~7.4). Acute horizontal brain slices were generated using a vibratome (VT1000S, Leica) at a thickness of 400 μm. Following brain cutting, slices were placed in a recovery chamber for 30 minutes in artificial cerebrospinal fluid (ACSF) at 35°C containing (in mM): 119 NaCl, 2.5 KCl, 26.2 NaHCO_3_, 1 NaH_2_PO_4_, 11 Glucose, 1.3 MgSO_4_ (gassed with 95% O_2_, 5% CO_2_ pH ~7.4). After incubation, the recovery chamber with slices was placed at RT with constant 95% O_2_, 5% CO_2_ bubbling maintained. Field potentials were recorded from the stratum radiatum of CA1. Slices were perfused in the recording chamber with oxygenated ACSF solution kept at 30°C. The recording electrode (3–4 MΩ resistance) was backfilled with ACSF and lowered 50 μm into the stratum radiatum of CA1. The Schaffer collateral inputs to CA1 were stimulated with a concentric bipolar electrode (FHC) located 150 μm from the recording electrode. Input-output curves and maximal fEPSP slope were acquired with stimulus pulses elicited at an intensity range from 2.5 μA – 25 μA every 30 s with a 0.5 ms stimulus duration using a Model 2100 Isolated Pulse Stimulator (A-M Systems). We adjusted the stimulus intensity to 30% of the maximal fEPSP slope to record the 20 min baseline for LTP recordings. Following recording of the baseline, LTP was induced with a 100 Hz, 1 s stimulation. Field potentials were recorded for 60 min after LTP induction. Field EPSP slopes were normalized to the mean baseline LTP slope. Recordings were acquired with WinLTP software (version 1.11b, University of Bristol) using a Multiclamp 700B amplifier (Molecular Devices). Recordings and analyses were performed blind to genotype.

#### Statistical analysis

Data are presented as mean ± SEM unless otherwise indicated. Statistical analyses were performed using GraphPad Prism 10.0 or R software. Comparisons between two groups were analyzed by unpaired Student’s t-test unless otherwise indicated. Multiple group comparisons were performed using one-way or two-way ANOVA, followed by Tukey’s or Bonferroni’s post-hoc tests. For behavioral experiments, repeated measures ANOVA was used for learning curves. Input-output and fiber volley data were analyzed with two-way repeated measures ANOVA followed by Bonferroni post-hoc test. Statistical significance was set at P < 0.05.

## Supplementary Material

Supplementary Files

This is a list of supplementary files associated with this preprint. Click to download.

- FangetalSupplementaryFigure1.tif
- FangetalSupplementaryFigure2.tif
- FangetalSupplementaryFigure3.tif
- FangetalSupplementaryFigure4.tif
- FangetalSupplementaryFigure5.tif
- FangetalSupplementaryFigure6.tif
- SupplementalTablesall.docx
- FangetalSupplementaryFigure1.mp4
- ga.png

## Data Availability

Raw sequencing was deposited in the Gene Expression Omnibus (GEO) database under accession numbers GSE310261 for GeoMx and GSE310266 for 10X. The reference DOI for Zenodo is 10.5281/zenodo.17559507 with the processed data of the snRNAseq and spatial genomics. Mass spectrometry data were deposited in the MetaboLights repository. Raw data and complete MS data sets have been uploaded to the Mass Spectrometry Interactive Virtual Environment (MassIVE) repository, developed by the Center for Computational Mass Spectrometry at the University of California San Diego, and can be downloaded using the following link: MassIVE Private Dataset MS000099236. https://massive.ucsd.edu/ProteoSAFe/private-dataset.jsp?task=50834f7b89f04eba9668adee7a54676e *Username:*MSV000099236_reviewer*Password: winter*. All other data supporting the conclusions of this article are available from the corresponding author upon reasonable request.

## HIGHLIGHTS

- CD38 expression increases in the choroid plexus pericytes with aging.
- CD38 loss preserves NAD^+^ in the brain and CSF of aged mice.
- CD38 deficiency prevents choroid plexus senescence.
- CSF shows less inflammation and higher NAD^+^ levels.
- Hippocampus regains youthful synaptic gene expression.
- CD38 inhibitor NTX-748 boosts NAD^+^ and cognition.
- CD38 inhibition in aged mice shows restored LTP and memory
- CD38 inhibition restores choroid plexus–CSF–brain signaling.
